# A COMPARISON OF DIRECT AND INDIRECT STUDENT CAREGIVERS ON OUTLOOKS RELATED TO CAREGIVING AND SELF

**DOI:** 10.1093/geroni/igae098.1574

**Published:** 2024-12-31

**Authors:** Allyson Graf, Shelby Baker, Robin Bartlett

**Affiliations:** Northern Kentucky University, Highland Heights, Kentucky, United States; Northern Kentucky University, Highland Heights, Kentucky, United States; Northern Kentucky University, Highland Heights, Kentucky, United States

## Abstract

Studies have recently demonstrated the impact of caregiving on college students. Caregiving, however, is transitional and dynamic and this complexity is rarely represented. The current study sought to compare caregiving experiences (direct-current, direct-former, and indirect care support) on the perceived impact on views of caregiving, relationships, well-being, and aging. Among the sample of 230 students (78.7% undergraduates) with caregiving experience, 28.3% were current caregivers, 53.5% were former caregivers, and 78.6% provided indirect care support. Compared to those with indirect experience, those with direct experience more strongly agree that caregiving impacted their views of caregiving (p <.001) and their relationship with care recipient (p <.001). There was no difference between current and former caregivers. Participants were also asked about perceived positive and negative influences independently. Results were similar for positive influences with direct caregivers perceiving a more positive influence on their relationship with the care recipient than indirect caregivers (p =.001) but only current caregivers differed significantly from indirect caregivers in the positive influence on views of caregiving (p =.023). Trends did not hold for perceived negative influence; however, there was a significant difference between current and former caregivers on the negative influence on health (p =.042) such that current caregivers indicated a greater negative influence. These results indicate the importance of investigating diverse caregiving experiences among students and differentiating positive and negative influences with implications for how universities may support the different life circumstances of student caregivers.

